# Characteristics and outcomes of COVID-19 patients with COPD from the United States, South Korea, and Europe

**DOI:** 10.12688/wellcomeopenres.17403.2

**Published:** 2022-03-24

**Authors:** David Moreno-Martos, Katia Verhamme, Anna Ostropolets, Kristin Kostka, Talita Duarte-Sales, Daniel Prieto-Alhambra, Thamir M Alshammari, Heba Alghoul, Waheed-Ul-Rahman Ahmed, Clair Blacketer, Scott DuVall, Lana Lai, Michael Matheny, Fredrik Nyberg, Jose Posada, Peter Rijnbeek, Matthew Spotnitz, Anthony Sena, Nigam Shah, Marc Suchard, Seng Chan You, George Hripcsak, Patrick Ryan, Daniel Morales

**Affiliations:** 1 Population Health and Genomics, University of Dundee, Dundee, UK; 2 Medical Informatics, Erasmus MC, Rotterdam, The Netherlands; 3 Biomedical Informatics, Columbia University Medical Center, New York, USA; 4 Real World Solutions, IQVIA, Cambridge, MA, USA; 5 OHDSI Center at The Roux Institute, Northeastern University, Portland, ME, USA; 6 Fundació Institut Universitari per a la recerca a l’Atenció Primaria de Salut Jordi Gol i Gurina (IDIAPJGol), IDIAPJGol, Barcelona, Spain; 7 Nuffield Department of Orthopaedics, Rheumatology, and Musculoskeletal Sciences, University of Oxford, Oxford, UK; 8 College of Pharmacy, Riyadh Elm University, Riyadh, Saudi Arabia; 9 Faculty of Medicine, Islamic University of Gaza, Gaza, Palestinian Territory; 10 College of Medicine and Health, University of Exeter, Exeter, UK; 11 Janssen Research and Development, Janssen Research and Development, Titusville, NJ, USA; 12 VA Informatics and Computing Infrastructure, University of Utah, Salt Lake City, UT, USA; 13 Department of Medical Sciences, University of Manchester, Manchester, UK; 14 Geriatrics Research Education and Clinical Care Service & VINCI, Tennessee Valley Healthcare System VA, nashville, TN, USA; 15 Department of Biomedical Informatics, Vanderbilt University Medical Center, Nashville, TN, USA; 16 School of Public Health and Community Medicine, University of Gothenburg, Gothenburg, Sweden; 17 Department of Medicine, Stanford University, Redwood City, CA, USA; 18 Department of Biostatistics UCLA Fielding School of Public Health, University of California, Los Angeles, Los Angeles, CA, USA; 19 Department of Computational Medicine David Geffen School of Medicine at UCLA,, University of California, Los Angeles, Los Angeles, CA, USA; 20 Department of Preventive Medicine, Yonsei University, Seoul, South Korea; 21 Department of Public Health, University of Southern Denmark, Odense, Denmark

**Keywords:** COPD, SARS-CoV-2, coronavirus, COVID, epidemiology.

## Abstract

**Background**: Characterization studies of COVID-19 patients with chronic obstructive pulmonary disease (COPD) are limited in size and scope. The aim of the study is to provide a large-scale characterization of COVID-19 patients with COPD.

**Methods**: We included thirteen databases contributing data from January-June 2020 from North America (US), Europe and Asia. We defined two cohorts of patients with COVID-19 namely a ‘diagnosed’ and ‘hospitalized’ cohort. We followed patients from COVID-19 index date to 30 days or death. We performed descriptive analysis and reported the frequency of characteristics and outcomes among COPD patients with COVID-19.

**Results**: The study included 934,778 patients in the diagnosed COVID-19 cohort and 177,201 in the hospitalized COVID-19 cohort. Observed COPD prevalence in the diagnosed cohort ranged from 3.8% (95%CI 3.5-4.1%) in French data to 22.7% (95%CI 22.4-23.0) in US data, and from 1.9% (95%CI 1.6-2.2) in South Korean to 44.0% (95%CI 43.1-45.0) in US data, in the hospitalized cohorts. COPD patients in the hospitalized cohort had greater comorbidity than those in the diagnosed cohort, including hypertension, heart disease, diabetes and obesity. Mortality was higher in COPD patients in the hospitalized cohort and ranged from 7.6% (95%CI 6.9-8.4) to 32.2% (95%CI 28.0-36.7) across databases. ARDS, acute renal failure, cardiac arrhythmia and sepsis were the most common outcomes among hospitalized COPD patients.

**Conclusion**: COPD patients with COVID-19 have high levels of COVID-19-associated comorbidities and poor COVID-19 outcomes. Further research is required to identify patients with COPD at high risk of worse outcomes.

## Introduction

Severe acute respiratory syndrome coronavirus 2 (SARS-CoV-2) has infected over 200 million patients and resulted in more than 4.2 million deaths worldwide as of April 2021 ^1^. Coronavirus disease 2019 (COVID-19) can lead to severe lung injury and pneumonia, acute kidney injury, cardiovascular complications, and death. The symptoms and complications of COVID-19 have been compared to seasonal influenza resulting in national policy measures classifying chronic obstructive pulmonary disease (COPD) patients as high risk and advising them to take additional protective measures ^2^. The prevalence of identified COPD among COVID-19 patients has ranged from 0.8% to 38% in the literature depending upon the cohort studied ^3^. Whilst some studies suggest that the prevalence of COPD among COVID-19 patients may be lower than the prevalence of COPD in the general population COPD is still considered a risk factor for severe COVID-19 disease ^4^.

Estimates for the prevalence of COPD among COVID-19 patients typically come from small, single-centre hospitalised cohorts and examine a limited range of patient characteristics and outcomes ^3^. Larger comparisons from geographically diverse cohorts that also include patients with milder COVID-19 illness provide a more compelling picture and improve generalisability. Viral respiratory tract infections are common triggers for exacerbations resulting in increased morbidity and mortality yet it is uncertain how often people with COPD with COVID-19 present with exacerbations ^5, 6^.

The aim of this study was to perform a large-scale, federated network, descriptive characterization study reporting the demographics, comorbidities, and outcomes of COPD patients with COVID-19 at the point of diagnosis and hospitalisation. 

## Methods

### Ethical approval

All the data partners received Institutional Review Board (IRB) approval or exemption. STARR-OMOP had approval from IRB Panel #8 (RB-53248) registered to Leland Stanford Junior University under the Stanford Human Research Protection Program (HRPP). The use of VA data was reviewed by the Department of Veterans Affairs Central Institutional Review Board (IRB), was determined to meet the criteria for exemption under Exemption Category 4(3), and approved for Waiver of HIPAA Authorization. The research was approved by the Columbia University Institutional Review Board as an OHDSI network study. The use of SIDIAP was approved by the Clinical Research Ethics Committee of the IDIAPJGol (project code: 20/070-PCV). The use of CPRD was approved by the Independent Scientific Advisory Committee (ISAC) (protocol number 20_059RA2). The use of IQVIA OpenClaims and IPCI was exempted from IRB approval for COVID-19 research.

### Study design

The Characterizing Health Associated Risks and Your Baseline Disease In SARS-COV-2 (CHARYBDIS) study is a multinational cohort study using retrospective electronic health records and claims data on COVID-19 patients from three continents, the North America (US), Europe, and Asia ^7^. All data for were standardized to the Observational Medical Outcomes Partnership (OMOP) Common Data Model (CDM) ^8, 9^. The Charybdis protocol and source code is available via open access ( https://github.com/ohdsi-studies/Covid19CharacterizationCharybdis) ^10^.

### Data sources

Of the nineteen databases available on 28 ^th^ November 2020, 13 that had a minimum sample size of 140 COVID-19 patients with COPD were included. This minimum threshold was considered appropriate to estimate the prevalence of a previous condition or 30-day risk of an outcome affecting 10% of the study population. Supplementary Figure S1 presents the database selection process for this study ^11^. 

Data from the United States included: the University of Colorado Anschuz Medical Campus Health Data Compass (CU-AMC HDC), the Columbia University Irving Medical Center data warehouse (CUIMC), HealthVerity, Stanford Medicine Research Data Repository (STARR-OMOP), IQVIA Open Claims, Optum de-identified Electronic Health Record Dataset and the United States Department of Veterans Affairs (VA-OMOP). Data from South Korea included the Health Insurance Review & Assessment Service (HIRA) database. Data from Europe included the Spanish Information System for Research in Primary Care (SIDIAP) database; the Dutch Integrated Primary Care Information (IPCI) database, LPD (Longitudinal Patients Database) France, LPD Italy and the UK Clinical Practice Research Datalink (CPRD). Further information about databases considered for inclusion is contained in Appendix 1 ^11^.

### Study participants and follow-up

*COVID-19 cohorts*: Two non-mutually exclusive cohorts were defined (Appendix 2) ^11^. COVID-19 patients in the *diagnosed* cohort were defined as patients having a clinical diagnosis and/or positive test for SARS-CoV-2 from outpatient or inpatient records. In the diagnosed cohort, the index date was the earliest date of COVID-19 diagnosis or a first positive test. COVID-19 patients in the *hospitalized* cohort were defined as patients with a hospitalization episode and a clinical diagnosis of COVID-19 or positive SARS-CoV-2 test within 21 days prior to admission and up to the end of hospitalization. This time window was chosen to include patients with a diagnosis prior to hospitalization and to allow for delays in recording of test results. In the hospitalized cohort, the index date was the day of hospitalization.

All patients were required to have at least a year of observation time prior to index date. Patients were followed from the index date to the earliest of the studied outcome, end of follow-up (30 days after index date), end of data capture, or death.

*COPD definition*: COPD was defined as either: a) an occurrence of a COPD diagnosis code any time on or before the COVID-19 index date or b) a prescription or administration of COPD medications within the year prior to index date in patients older than or equal to 55 years (Appendix 3) ^11^. We excluded patients with a diagnosis of asthma prior to the COPD diagnosis to avoid misclassification with asthma. Codes used to define these cohorts have been previously described ^12, 13^ and are included in the Appendix ^11^.

### Baseline characteristics

Conditions and procedures were identified within 1 to 365 to days prior to the index date using Systematized Nomenclature of Medicine (SNOMED CT) codes with all descendent codes included codes mapped from local source vocabularies. We report pre-specified demographics and conditions related to COPD and COVID-19. Other conditions analysed as part of the larger CHARYBDIS project are reported here ( https://data.ohdsi.org/Covid19CharacterizationCharybdis/).

COPD exacerbation was defined by a COPD exacerbation code at index date in databases with disease codes for exacerbation. The following medications were identified within 1 to 30 days prior to index date to characterise how patients were recently managed prior to the COVID-19 index date: systemic corticosteroids, inhaled corticosteroids (ICS), short-acting beta2-agonists (SABA), long-acting beta2-agonists (LABA), short-acting muscarinic antagonists (SAMA), long-acting antimuscarinic antagonists (LAMA), methylxanthines, mucolytics, oxygen therapy, antibiotics (beta-lactam penicillins, macrolides, fluoroquinolones), acetaminophen, nonsteroidal anti-inflammatory drugs (NSAIDs), and opioids. Medication use was calculated using drug eras that began starting on the date of the first drug exposure and ended on the observed end date of the last concatenated medication record, with a grace period of 30 days between medication records which allowed for sequential medication records to be considered as a continuous drug era.

### Outcomes

We identified the following outcomes within 30 days following the index date: death, use of intensive services (identified as a recorded invasive mechanical ventilation and/or a tracheostomy and/or extracorporeal membrane oxygenation procedure), acute respiratory distress syndrome (ARDS), acute renal failure syndrome (ARFS), cardiac arrhythmia, heart failure, pulmonary oedema, myocardial infarction, sepsis, bleeding, venous thromboembolism (VTE), pulmonary embolism (PE) and stroke (ischaemic and haemorrhagic).

### Analysis

A common analytical code for CHARYBDIS was run locally in each database. Only aggregate results from each database were then shared. We report the number and proportion by socio-demographics, history of comorbidities, and commonly used medications in each population with 95% confidence intervals (CI) calculated using the Wilson score method. Standardised mean differences (SMD) were calculated to aid comparison between study cohorts. We used R version 3.6.0 for data visualization. All the data partners obtained Institutional Review Board (IRB) approval or exemption to conduct this study, as required.

## Results

### Prevalence of COPD

The study included 934,778 COVID-19 patients in the diagnosed cohort (84.0% from US and 16.0% from European databases) and 177,201 COVID-19 patients in the hospitalized cohort (87.5% from US, 8.8% from European and 3.7% from South Korean databases). The observed prevalence of COPD in the diagnosed cohort ranged from 3.8% (95%CI 3.5-4.1) in data from France to 22.7% (95%CI 22.4-23.0) in the US (overall median 7.7%) ( Table 1). The observed prevalence of COPD in COVID-19 patients in the hospitalized cohort ranged from 1.9% (95%CI 1.6-2.2) in data from South Korea to 44.0% (95%CI 43.1-45.0) in the US (overall median 20.9%). Among databases contributing to both COVID-19 cohorts, the prevalence of COPD was greater in hospitalized than in the diagnosed COVID-19 cohorts. COPD exacerbation at presentation ranged from <0.8% to 6.4% (95%CI 4.8-8.5) in the *diagnosed* cohort (median 4.1%), and from 1.3% (95%CI 0.6-2.8) to 12.0% (95%CI 8.8-16.2) amongst those in the *hospitalized* cohort (median 7.7%) ( Table 2).

**Table 1.** Prevalence of COPD in COVID-19 patients from 26 database cohorts in 7 countries.

**Cohort**	**US** **CU-AMC**	**US** **CUIMC**	**US** **HealthVerity**	**US** **IQVIA** **OpenClaims**	**US** **Optum EHR**	**US** **STARR-** **OMOP**	**US** **VA-OMOP**	**KR** **HIRA**	**UK** **CPRD**	**NL** **IPCI**	**ES** **SIDIAP**	**FR** **LPD** **France**	**IT** **LPD** **Italy**
**Diagnosed ** **COVID-19**	7270	8519	114173	466191	129512	3328	55557	-	3372	3047	122141	17180	4488
**COPD (n)**	692	770	6500	68262	11056	218	12610	-	261	200	15803	648	340
**Prevalence ** **(%), 95%CI**	9.5 8.9-10.2	9.0 8.4-9.7	5.7 5.6-5.8	14.6 14.5-14.7	8.5 8.4-8.7	6.6 5.8-7.4	22.7 22.4-23.0	-	7.7 6.9-8.7	6.6 5.7-7.5	12.9 12.8-13.1	3.8 3.5-4.1	7.6 6.8-8.4
**Hospitalized ** **COVID-19**	1434	2600	7581	133091	22024	-	10471	7599	-	-	18202	-	-
**COPD (n)**	299	447	1649	30917	4504	-	4611	145	-	-	4843	-	-
**Prevalence ** **(%), 95%CI**	20.9 18.8-23.0	17.2 15.8-18.7	21.8 20.8-22.7	23.2 23.0-23.5	20.5 19.9-21.0	-	44.0 43.1-45.0	1.9 1.6-2.2	-	-	26.6 26.0-27.3	-	-

Grey =US database. Pink=South Korean database. Yellow=European database. US=United States. KR=South Korea. UK=United Kingdom. NL=Netherlands. ES=Spain. (n) = number. NA= not applicable for that cohort.

**Table 2.** Prevalence of COPD exacerbation in COVID-19 patients.

Cohort	**US** **CU-AMC**	**US** **CUIMC**	**US** **HealthVerity**	**US** **IQVIA** **OpenClaims**	**US** **Optum** **EHR**	**US** **STARR-** **OMOP**	**US** **VA-OMOP**	**KR** **HIRA**	**UK** **CPRD**	**ES** **SIDIAP**	**FR** **LPD** **France**
Diagnosed COVID-19 Prevalence (%), 95%CI	6.4 4.8-8.5	1.9 1.1-3.1	3.7 3.3-4.2	5.0 4.8-5.2	6.2 5.8-6.7	4.1 2.2-7.6	2.9 2.6-3.2	-	<1.9	1.8 1.6-2.0	<0.8
Hospitalized COVID-19 Prevalence (%), 95%CI	12.0 8.8-16.2	1.3 0.6-2.8	8.6 7.3-10.1	7.7 7.4-8.0	10.8 9.9-11.7	-	5.5 4.9-6.2	<3.4	-	3.2 2.7-3.7	-

*No data on COPD exacerbations recorded for IPCI and LDP Italy. Grey =US database. Pink=South Korean database. Yellow=European database. US=United States. KR=South Korea. UK=United Kingdom. NL=Netherlands. ES=Spain.

### Demographics

In the *hospitalized* cohort, COPD patients with COVID-19 were more commonly male (range 46.8% to 58.5%, overall median 54.5%) (Supplementary Table S1) ^11^. However, there was less consistent sex difference amongst patients in the *diagnosed* cohort (Supplementary Table S2) ^11^. Whilst in VA-OMOP 96.7% of hospitalized patients and 94.6% of diagnosed patients were male, this was expected due to the population demographics with data predominantly originating from male veterans. In both cohorts, COPD patients with COVID-19 had a similar distribution of age and were more commonly older than 65 years (Supplementary Figure S2 and S3) ^11^.

### Baseline comorbidities

**Figure 1.**
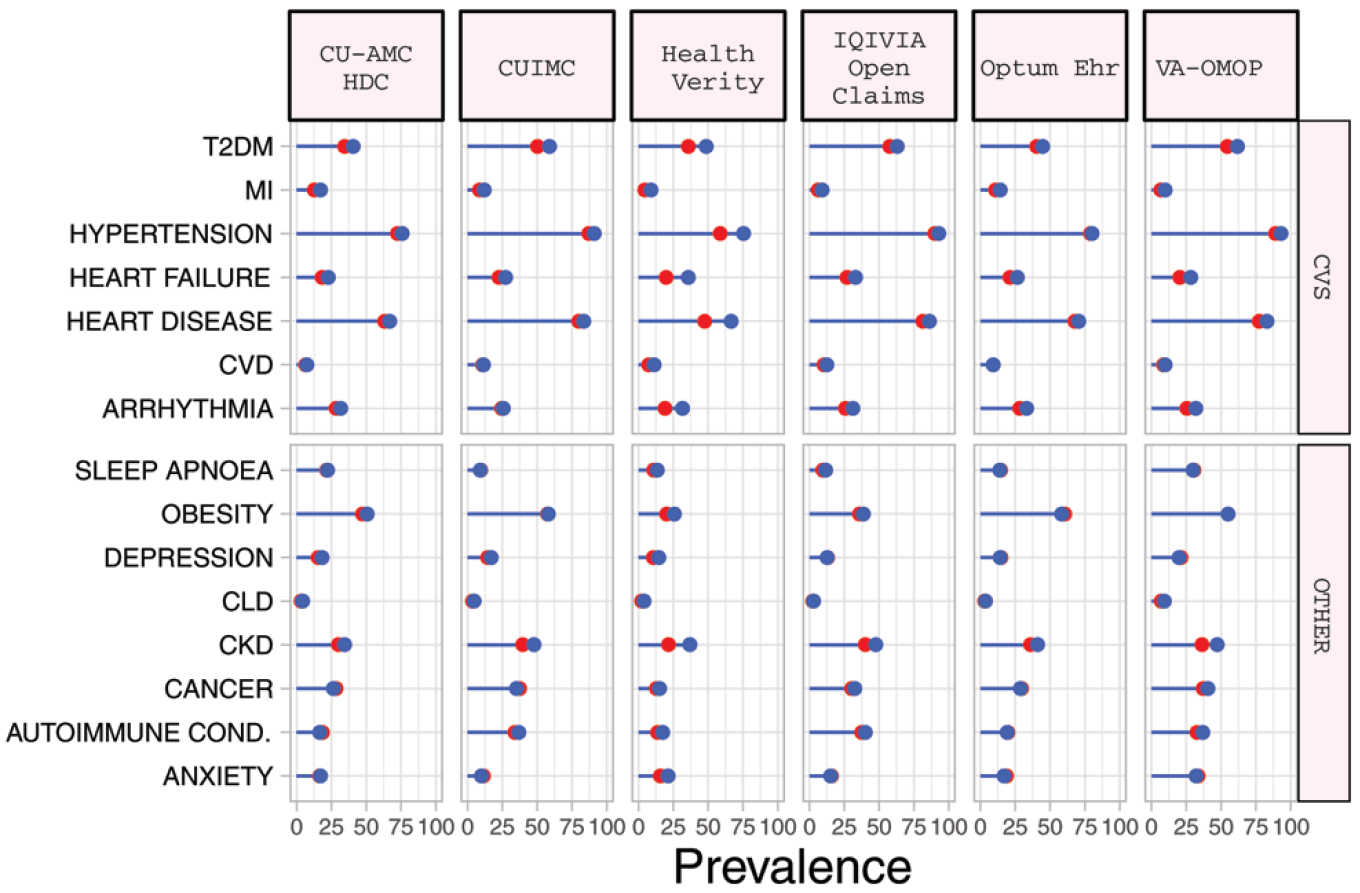
Prevalence of comorbidities among COPD patients with COVID-19 who have been diagnosed (red) and hospitalized (blue). *Databases contributing patients to both the diagnosed and hospitalized cohorts. T2DM=Type 2 diabetes mellitus. MI=myocardial infarction. CVD=Cerebrovascular disease. CLD=chronic liver disease. CKD=chronic kidney disease. Autoimmune Cond.=autoimmune conditions. CVS=Cardiovascular.

In the *diagnosed* COPD cohort, the most prevalent comorbidities included obesity (median 49.1%), cardiovascular disease (median 63.2%), hypertension (median 72.4%), chronic kidney disease (CKD) (median 29.8%) and type 2 diabetes mellitus (T2DM) (median 35.9%) ( Table 3). Compared to those in the diagnosed cohort, the *hospitalized* COPD cohort had a greater prevalence of cardiovascular comorbidities, T2DM and CKD although these differences were typically modest ( Table 4, Figure 1, Supplementary Figure S3) ^11^.

**Table 3.** Prevalence of comorbidity in COPD patients in the diagnosed COVID-19 cohort.

	**CU-AMC HDC** **% (95%CI)**	**CUIMC** **% (95%CI)**	**HealthVerity** **% (95%CI)**	**IQVIA-OpenClaims** **% (95%CI)**	**OptumEhr** **% (95%CI)**	**STARR-OMOP** **% (95%CI)**	**VA-OMOP** **% (95%CI)**	**CPRD** **% (95%CI)**	**IPCI** **% (95%CI)**	**SIDIAP** **% (95%CI)**	**LPD-FRANCE** **% (95%CI)**	**LPDItaly** **% (95%CI)**
**Anxiety**	16.6 (14.0-19.6)	11.4 (9.3-13.8)	15.7 (14.8-16.6)	15.9 (15.6-16.2)	18.6 (17.9-19.3)	21.1 (16.2-27.0)	33.5 (32.7-34.3)	<1.9	8.0 (5.0-12.6)	26.3 (25.6-27.0)	11.6 (9.4-14.3)	10.3 (7.5-14.0)
**Atrial fibrillation**	16.3 (13.7-19.2)	16.2 (13.8-19.0)	13.8 (13.0-14.7)	17.5 (17.2-17.8)	17.0 (16.3-17.7)	17.4 (12.9-23.0)	17.9 (17.2-18.6)	4.2 (2.4-7.4)	9.5 (6.2-14.4)	15.1 (14.5-15.7)	<0.8	12.4 (9.3-16.3)
**Autoimmune condition**	18.6 (15.9-21.7)	33.9 (30.6-37.3)	13.7 (12.9-14.6)	37.4 (37.0-37.8)	19.9 (19.2-20.6)	18.8 (14.2-24.5)	32.6 (31.8-33.4)	13.8 (10.1-18.5)	27.5 (21.8-34.1)	12.0 (11.5-12.5)	18.5 (15.7-21.7)	22.4 (18.3-27.1)
**Bronchiectasis**	<1.4	2.9 (1.9-4.3)	1.1 (0.9-1.4)	1.4 (1.3-1.5)	1.5 (1.3-1.7)	5.5 (3.2-9.4)	0.8 (0.7-1.0)	<1.9	<2.5	4.1 (3.8-4.4)	1.2 (0.6-2.4)	<1.5
Cancer *	28.2 (25.0-31.7)	37.4 (34.1-40.9)	12.9 (12.1-13.7)	30.3 (30.0-30.6)	29.5 (28.7-30.4)	47.2 (40.7-53.8)	37.3 (36.5-38.1)	14.9 (11.1-19.7)	28.0 (22.2-34.6)	19.0 (18.4-19.6)	9.6 (7.6-12.1)	27.1 (22.7-32.1)
**Cardiac arrhythmia**	28.5 (25.3-32.0)	24.4 (21.5-27.6)	18.9 (18.0-19.9)	25.6 (25.3-25.9)	28.0 (27.2-28.8)	28.0 (22.5-34.3)	25.6 (24.9-26.4)	4.6 (2.6-7.9)	11.0 (7.4-16.1)	26.4 (25.7-27.1)	5.7 (4.2-7.8)	19.1 (15.3-23.6)
**Cerebrovascular disease**	6.6 (5.0-8.7)	10.8 (8.8-13.2)	7.3 (6.7-8.0)	10.4 (10.2-10.6)	9.0 (8.5-9.6)	7.3 (4.5-11.5)	8.5 (8.0-9.0)	4.6 (2.6-7.9)	6.5 (3.8-10.8)	4.0 (3.7-4.3)	4.0 (2.7-5.8)	10.9 (8.0-14.7)
**Chronic Liver Disease**	3.0 (2.0-4.6)	3.6 (2.5-5.2)	2.3 (2.0-2.7)	2.5 (2.4-2.6)	3.2 (2.9-3.5)	9.6 (6.4-14.2)	7.2 (6.8-7.7)	-	-	2.2 (2.0-2.4)	<0.8	3.5 (2.0-6.0)
CKD *	29.8 (26.5-33.3)	39.5 (36.1-43.0)	21.6 (20.6-22.6)	40.0 (39.6-40.4)	36.0 (35.1-36.9)	33.9 (27.9-40.4)	36.3 (35.5-37.1)	26.1 (21.1-31.8)	23.0 (17.7-29.3)	23.0 (22.4-23.7)	3.5 (2.3-5.2)	11.2 (8.3-15.0)
**Depression**	15.6 (13.1-18.5)	14.3 (12.0-17.0)	10.7 (10.0-11.5)	12.8 (12.6-13.1)	14.9 (14.2-15.6)	19.3 (14.6-25.0)	21.2 (20.5-21.9)	6.9 (4.4-10.6)	3.0 (1.4-6.4)	18.2 (17.6-18.8)	11.9 (9.6-14.6)	8.8 (6.2-12.3)
Heart disease *	63.2 (59.5-66.7)	80.0 (77.0-82.7)	47.4 (46.2-48.6)	81.4 (81.1-81.7)	67.7 (66.8-68.6)	65.6 (59.1-71.6)	77.2 (76.5-77.9)	44.8 (38.9-50.9)	49.5 (42.6-56.4)	43.7 (42.9-44.5)	18.7 (15.9-21.9)	46.5 (41.3-51.8)
**Heart failure**	18.2 (15.5-21.2)	22.6 (19.8-25.7)	19.9 (19.0-20.9)	26.8 (26.5-27.1)	21.2 (20.4-22.0)	19.7 (15.0-25.5)	20.4 (19.7-21.1)	4.6 (2.6-7.9)	8.5 (5.4-13.2)	12.3 (11.8-12.8)	2.0 (1.2-3.4)	5.3 (3.4-8.2)
Hypertension *	72.4 (69.0-75.6)	86.9 (84.3-89.1)	58.8 (57.6-60.0)	90.0 (89.8-90.2)	79.1 (78.3-79.8)	75.2 (69.1-80.5)	89.1 (88.5-89.6)	30.7 (25.4-36.5)	49.5 (42.6-56.4)	39.0 (38.2-39.8)	43.1 (39.3-46.9)	66.8 (61.6-71.6)
**Interstitial lung disease**	1.6 (0.9-2.8)	2.5 (1.6-3.9)	2.1 (1.8-2.5)	2.6 (2.5-2.7)	2.4 (2.1-2.7)	4.1 (2.2-7.6)	1.6 (1.4-1.8)	<1.9	-	0.5 (0.4-0.6)	<0.8	<1.5
**Myocardial infarction**	12.6 (10.3-15.3)	8.6 (6.8-10.8)	4.6 (4.1-5.1)	6.3 (6.1-6.5)	10.7 (10.1-11.3)	10.6 (7.2-15.4)	6.7 (6.3-7.1)	<1.9	6.0 (3.5-10.2)	2.7 (2.5-3.0)	<0.8	3.5 (2.0-6.0)
**Obesity**	47.5 (43.8-51.2)	57.3 (53.8-60.8)	20.1 (19.1-21.1)	35.8 (35.4-36.2)	60.7 (59.8-61.6)	49.1 (42.5-55.7)	55.2 (54.3-56.1)	50.6 (44.6-56.6)	33.0 (26.9-39.8)	52.1 (51.3-52.9)	14.4 (11.9-17.3)	25.6 (21.2-30.5)
**Sleep apnea**	21.5 (18.6-24.7)	9.6 (7.7-11.9)	10.9 (10.2-11.7)	9.8 (9.6-10.0)	14.4 (13.8-15.1)	23.4 (18.3-29.4)	30.6 (29.8-31.4)	<1.9	<2.5	6.0 (5.6-6.4)	1.2 (0.6-2.4)	2.1 (1.0-4.2)
T2DM *	34.5 (31.0-38.1)	50.4 (46.9-53.9)	35.9 (34.7-37.1)	57.6 (57.2-58.0)	40.6 (39.7-41.5)	40.8 (34.5-47.4)	54.5 (53.6-55.4)	24.9 (20.0-30.5)	34.0 (27.8-40.8)	21.6 (21.0-22.2)	18.2 (15.4-21.4)	20.6 (16.6-25.2)

*Prevalent conditions measured any time in the past, others within the previous year. Grey =US database. Pink=South Korean database. Yellow=European database. CKD=Chronic kidney disease. T2DM=type 2 diabetes mellitus.

**Table 4.** Prevalence of comorbidity in hospitalized COVID-19 patients with COPD.

	**CU-AMC HDC** **% (95%CI)**	**CUIMC** **% (95%CI)**	**HealthVerity** **% (95%CI)**	**IQVIA-OpenClaims** **% (95%CI)**	**OptumEhr** **% (95%CI)**	**VA-OMOP** **% (95%CI)**	**HIRA** **% (95%CI)**	**SIDIAP** **% (95%CI)**
**Anxiety**	17.1 (13.3-21.8)	10.1 (7.6-13.2)	21.4 (19.5-23.4)	15.3 (14.9-15.7)	17.0 (15.9-18.1)	32.3 (31.0-33.7)	20.0 (14.3-27.3)	23.3 (22.1-24.5)
**Atrial fibrillation**	19.1 (15.0-23.9)	17.0 (13.8-20.8)	23.7 (21.7-25.8)	20.5 (20.0-21.0)	20.0 (18.9-21.2)	22.3 (21.1-23.5)	<3.4	16.7 (15.7-17.8)
**Autoimmune condition**	16.7 (12.9-21.3)	36.7 (32.4-41.3)	17.3 (15.6-19.2)	40.0 (39.5-40.6)	19.1 (18.0-20.3)	36.9 (35.5-38.3)	20.7 (14.9-28.0)	12.7 (11.8-13.7)
**Bronchiectasis**	<3.3	3.4 (2.1-5.5)	1.6 (1.1-2.3)	1.7 (1.6-1.8)	1.5 (1.2-1.9)	0.8 (0.6-1.1)	<3.4	5.0 (4.4-5.7)
Cancer *	26.4 (21.7-31.7)	35.3 (31.0-39.8)	15.1 (13.5-16.9)	32.5 (32.0-33.0)	28.8 (27.5-30.1)	40.3 (38.9-41.7)	20.0 (14.3-27.3)	22.4 (21.2-23.6)
**Cardiac arrhythmia**	31.8 (26.8-37.3)	25.5 (21.7-29.7)	31.6 (29.4-33.9)	31.1 (30.6-31.6)	33.3 (31.9-34.7)	31.9 (30.6-33.3)	9.7 (5.9-15.6)	29.6 (28.3-30.9)
**Cerebrovascular disease**	7.4 (4.9-10.9)	11.4 (8.8-14.7)	11.0 (9.6-12.6)	12.4 (12.0-12.8)	9.2 (8.4-10.1)	9.8 (9.0-10.7)	13.1 (8.6-19.6)	3.8 (3.3-4.4)
**Chronic Liver Disease**	4.3 (2.5-7.2)	4.7 (3.1-7.1)	4.1 (3.2-5.2)	3.1 (2.9-3.3)	3.4 (2.9-4.0)	9.1 (8.3-10.0)	10.3 (6.3-16.3)	1.9 (1.6-2.3)
CKD *	34.4 (29.2-40.0)	47.7 (43.1-52.3)	36.7 (34.4-39.1)	47.7 (47.1-48.3)	40.8 (39.4-42.2)	47.3 (45.9-48.7)	17.2 (11.9-24.2)	25.2 (24.0-26.4)
**Depression**	18.4 (14.4-23.2)	17.0 (13.8-20.8)	14.6 (13.0-16.4)	12.7 (12.3-13.1)	14.1 (13.1-15.2)	19.7 (18.6-20.9)	6.9 (3.8-12.2)	14.8 (13.8-15.8)
Heart disease *	66.9 (61.4-72.0)	83.4 (79.7-86.6)	66.3 (64.0-68.5)	86.1 (85.7-86.5)	70.7 (69.3-72.0)	83.0 (81.9-84.1)	38.6 (31.1-46.7)	49.6 (48.2-51.0)
**Heart failure**	22.7 (18.3-27.8)	27.1 (23.2-31.4)	35.8 (33.5-38.1)	33.2 (32.7-33.7)	26.4 (25.1-27.7)	28.4 (27.1-29.7)	15.2 (10.3-21.9)	14.0 (13.1-15.0)
Hypertension *	75.9 (70.7-80.4)	90.8 (87.8-93.1)	75.3 (73.2-77.3)	93.0 (92.7-93.3)	79.9 (78.7-81.0)	93.0 (92.2-93.7)	55.9 (47.8-63.7)	41.1 (39.7-42.5)
**Interstitial lung disease**	<3.3	3.1 (1.8-5.1)	3.5 (2.7-4.5)	4.6 (4.4-4.8)	3.2 (2.7-3.8)	2.1 (1.7-2.6)	<3.4	0.8 (0.6-1.1)
**Myocardial infarction**	17.1 (13.3-21.8)	11.9 (9.2-15.2)	8.8 (7.5-10.3)	9.1 (8.8-9.4)	14.3 (13.3-15.3)	9.8 (9.0-10.7)	<3.4	3.6 (3.1-4.2)
**Obesity**	50.5 (44.9-56.1)	58.2 (53.6-62.7)	25.7 (23.6-27.9)	38.8 (38.3-39.3)	58.3 (56.9-59.7)	55.0 (53.6-56.4)	-	57.7 (56.3-59.1)
**Sleep apnea**	22.1 (17.8-27.1)	9.2 (6.9-12.2)	13.5 (11.9-15.2)	11.6 (11.2-12.0)	13.9 (12.9-14.9)	29.7 (28.4-31.0)	-	8.3 (7.6-9.1)
T2DM *	40.5 (35.1-46.2)	58.8 (54.2-63.3)	48.6 (46.2-51.0)	63.0 (62.5-63.5)	44.6 (43.1-46.1)	61.6 (60.2-63.0)	57.2 (49.1-65.0)	25.6 (24.4-26.9)

*Prevalent conditions measured any time in the past, others within the previous year. Grey =US database. Pink=South Korean database. Yellow=European database. CKD=Chronic kidney disease. T2DM=type 2 diabetes mellitus.

### Medication use

**Figure 2.**
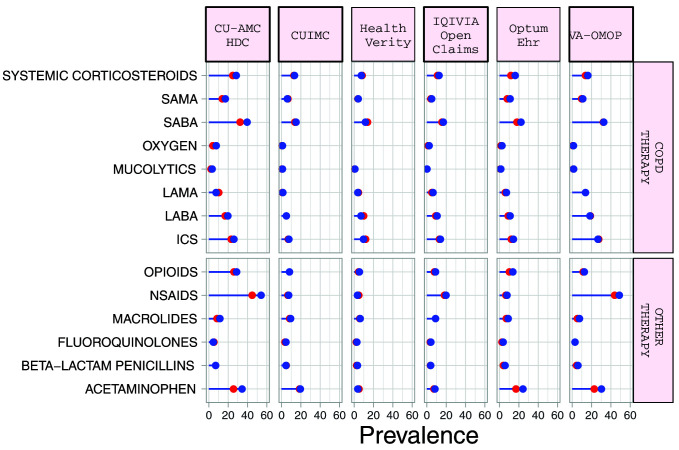
Prevalence of treatments among COPD patients with COVID-19 who have been diagnosed (red) and hospitalized (blue). *Databases contributing patients to both the diagnosed and hospitalized cohorts. SAMA=short-acting muscarinic antagonist. SABA=short-acting beta2-agonist. LAMA=Long-acting muscarinic antagonist. LABA=Long-acting beta2-agonist. ICS=inhaled corticosteroids. NSAIDs=non-steroidal anti-inflammatory drugs.

Systemic corticosteroid use in the 30 days prior to index date ranged from 5.1% (95%CI 3.7-7.1) to 26.4% (95%CI 21.4-32.1) in the *diagnosed* cohort, and from 7.5% (95%CI 6.3-8.9) to 28.4% (95%CI 23.6-33.8) in the *hospitalized* cohort ( Table 5 and Supplementary Table S3) ^11^. Corresponding numbers for inhaled corticosteroid (ICS) use ranged from 5.3% (95%CI 3.4-8.27.7) to 44.8% (95%CI 38.9-50.9), and from 7.6% (95%CI 5.5-10.4) to 33.7% (95%CI 32.4-35.0) respectively ( Figure 2). LABA use was more frequent than LAMA use. Macrolides were the most commonly prescribed antibiotics in the 30 days before index date in the US, French, and Italian databases while beta-lactam penicillins were more frequently prescribed in the South Korean, UK, Spanish and Dutch databases. Acetaminophen and NSAIDs were commonly prescribed to COPD patients, with use of both being more prevalent in the *hospitalized* cohort.

**Table 5.** Prevalence of treatments in patients with COPD in the 30 days before COVID-19 hospitalization.

	CU-AMC HDC % (95%CI)	CUIMC % (95%CI)	HealthVerity % (95%CI)	IQVIA- OpenClaims % (95%CI)	OptumEhr % (95%CI)	VA-OMOP % (95%CI)	HIRA % (95%CI)	SIDIAP % (95%CI)
**Systemic ** ** corticosteroids**	28.4 (23.6-33.8)	13.4 (10.5-16.9)	7.5 (6.3-8.9)	12.7 (12.3-13.1)	16.1 (15.1-17.2)	16.1 (15.1-17.2)	23.4 (17.2-30.9)	17.9 (16.9-19.0)
**Inhaled ** ** corticosteroid**	25.8 (21.2-31.0)	7.6 (5.5-10.4)	9.6 (8.3-11.1)	14.0 (13.6-14.4)	14.4 (13.4-15.5)	26.5 (25.2-27.8)	<6.8	33.7 (32.4-35.0)
**SABA**	33.4 (28.3-38.9)	13.0 (10.2-16.4)	9.5 (8.2-11.0)	14.4 (14.0-14.8)	20.6 (19.4-21.8)	26.9 (25.6-28.2)	<3.4	14.3 (13.3-15.3)
**SAMA**	17.1 (13.3-21.8)	6.0 (4.2-8.6)	4.1 (3.2-5.2)	4.9 (4.7-5.1)	11.1 (10.2-12.0)	10.8 (9.9-11.7)	<3.4	22.4 (21.2-23.6)
**LABA**	19.7 (15.6-24.6)	5.1 (3.4-7.6)	7.1 (6.0-8.4)	10.6 (10.3-10.9)	10.9 (10.0-11.8)	18.4 (17.3-19.5)	4.1 (1.9-8.7)	30.4 (29.1-31.7)
**LAMA**	7.4 (4.9-10.9)	1.3 (0.6-2.8)	3.8 (3.0-4.8)	6.4 (6.1-6.7)	7.2 (6.5-8.0)	13.8 (12.8-14.8)	<3.4	19.3 (18.2-20.4)
**Mucolytics**	<3.3	<1.1	0.8 (0.5-1.4)	0.2 (0.2-0.3)	1.3 (1.0-1.7)	1.5 (1.2-1.9)	71.0 (63.1-77.8)	12.4 (11.5-13.4)
**Beta-lactam** ** penicillins**	7.0 (4.6-10.5)	4.7 (3.1-7.1)	3.3 (2.5-4.3)	4.0 (3.8-4.2)	5.9 (5.2-6.6)	5.9 (5.3-6.6)	13.1 (8.6-19.6)	11.7 (10.8-12.6)
**Fluoroquinolones**	4.7 (2.8-7.7)	4.7 (3.1-7.1)	2.8 (2.1-3.7)	4.3 (4.1-4.5)	3.7 (3.2-4.3)	2.8 (2.4-3.3)	9.7 (5.9-15.6)	10.0 (9.2-10.9)
**Macrolides**	11.4 (8.3-15.5)	9.4 (7.0-12.5)	6.1 (5.0-7.4)	8.9 (8.6-9.2)	9.1 (8.3-10.0)	7.5 (6.8-8.3)	9.7 (5.9-15.6)	10.6 (9.8-11.5)
**Acetaminophen**	34.4 (29.2-40.0)	19.5 (16.1-23.4)	3.9 (3.1-5.0)	8.4 (8.1-8.7)	24.2 (23.0-25.5)	30.3 (29.0-31.6)	45.5 (37.6-53.6)	60.4 (59.0-61.8)
**NSAIDs**	54.2 (48.5-59.8)	7.4 (5.3-10.2)	3.5 (2.7-4.5)	19.7 (19.3-20.2)	7.8 (7.0-8.6)	48.8 (47.4-50.2)	27.6 (21.0-35.4)	9.2 (8.4-10.1)
**Opioids**	28.8 (24.0-34.2)	8.3 (6.1-11.2)	5.3 (4.3-6.5)	9.0 (8.7-9.3)	13.7 (12.7-14.7)	12.5 (11.6-13.5)	48.3 (40.3-56.4)	17.6 (16.6-18.7)
**Oxygen**	7.7 (5.2-11.3)	<1.1	2.8 (2.1-3.7)	2.4 (2.2-2.6)	2.5 (2.1-3.0)	1.4 (1.1-1.8)	4.8 (2.3-9.6)	<0.1

Grey =US database. Pink=South Korean database. Yellow=European database. SABA=short-acting beta-agonist. SAMA=short-acting muscarinic-antagonist. LABA=long-acting beta-agonist. LAMA=Long-acting muscarinic-antagonist. NSAIDs=non-steroidal anti-inflammatory drugs.

### Outcomes

**Figure 3.**
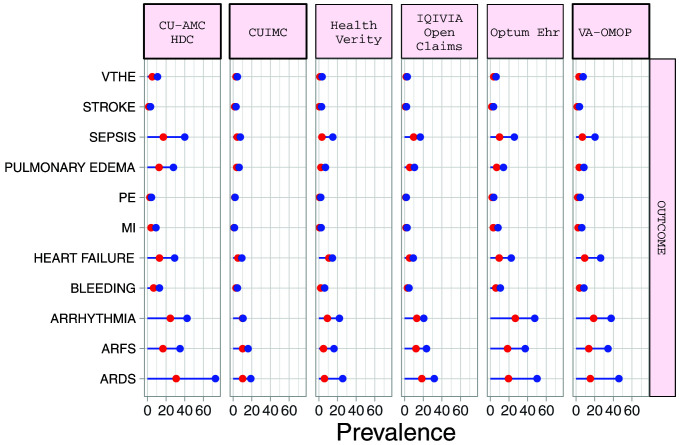
Prevalence of outcomes among COPD patients with COVID-19 who have been diagnosed (red) and hospitalized (blue). *Databases contributing patients to both the diagnosed and hospitalized cohorts. VTHE=venous thromboembolism. PE=pulmonary edema. MI=myocardial infarction. ARFS=Acute renal failure syndrome. ARDS=Acute respiratory distress syndrome.

The proportion of hospitalized COVID-19 patients requiring intensive services varied from 5.5% (95%CI 4.5-6.7) to 30.8% (95%CI 25.8-36.2) (median 17.7%). Amongst hospitalized COPD patients with COVID-19, the most common 30-day outcomes included ARDS (median 45.9%), ARFS (median 45.9%), cardiac arrhythmia (median 29.6%), heart failure (median 14.6%) and sepsis (median 16.9%). Outcomes were more common in the hospitalised cohort than the diagnosed patients ( Figure 3). ( Table 6 and Supplementary Table S4) ^11^. Among COPD patients with COVID-19 in the hospitalized cohort, 30-day mortality ranged from 7.6% (95%CI 6.9-8.4) to 32.2% (95%CI 28.0-36.7) (medium 21.4%), whilst in the diagnosed cohort 30-day mortality ranged from 3.7% (95%CI 3.4-4.1) to 24.9% (95%CI 20.0-30.5) (median 15.6%) ( Table 7).

**Table 6.** Prevalence of outcomes in hospitalized COVID-19 patients with COPD.

	CU-AMC HDC % (95%CI)	CUIMC % (95%CI)	HealthVerity % (95%CI)	IQVIA- OpenClaims % (95%CI)	OptumEhr % (95%CI)	VA-OMOP % (95%CI)	HIRA % (95%CI)
**Intensive** ** services**	30.8 (25.8-36.2)	NA	5.5 (4.5-6.7)	9.9 (9.6-10.2)	17.7 (16.6-18.8)	18.4 (17.3-19.5)	9.7 (5.9-15.6)
**ARDS**	72.9 (67.6-77.6)	19.0 (15.6-22.9)	25.6 (23.5-27.8)	31.7 (31.2-32.2)	50.2 (48.7-51.7)	45.9 (44.5-47.3)	<3.4
**ARFS**	35.1 (29.9-40.7)	16.1 (13.0-19.8)	16.3 (14.6-18.2)	23.5 (23.0-24.0)	37.4 (36.0-38.8)	34.4 (33.0-35.8)	<3.4
**Cardiac** ** arrhythmia**	48.2 (42.6-53.8)	14.8 (11.8-18.4)	15.5 (13.8-17.3)	14.9 (14.5-15.3)	37.4 (36.0-38.8)	37.8 (36.4-39.2)	7.6 (4.3-13.1)
**Heart ** **failure**	29.1 (24.2-34.5)	9.4 (7.0-12.5)	14.6 (13.0-16.4)	9.3 (9.0-9.6)	22.4 (21.2-23.6)	26.4 (25.1-27.7)	4.8 (2.3-9.6)
**Pulmonary ** **edema**	27.8 (23.0-33.1)	6.5 (4.6-9.2)	7.0 (5.9-8.3)	10.5 (10.2-10.8)	14.2 (13.2-15.2)	8.3 (7.5-9.1)	4.1 (1.9-8.7)
**Myocardial** ** infarction**	9.0 (6.3-12.8)	1.3 (0.6-2.8)	2.4 (1.8-3.3)	2.6 (2.4-2.8)	8.3 (7.5-9.1)	6.1 (5.4-6.8)	<3.4
**Sepsis**	40.1 (34.7-45.8)	7.6 (5.5-10.4)	14.9 (13.3-16.7)	16.9 (16.5-17.3)	25.8 (24.5-27.1)	20.3 (19.2-21.5)	5.5 (2.8-10.5)
**Bleeding**	13.0 (9.7-17.3)	4.5 (2.9-6.8)	6.2 (5.1-7.5)	4.8 (4.6-5.0)	10.9 (10.0-11.8)	8.3 (7.5-9.1)	6.2 (3.3-11.4)
**VThE**	10.7 (7.7-14.7)	4.5 (2.9-6.8)	3.5 (2.7-4.5)	2.8 (2.6-3.0)	6.3 (5.6-7.0)	7.6 (6.9-8.4)	-
**PE**	4.3 (2.5-7.2)	2.0 (1.0-3.8)	2.0 (1.4-2.8)	1.9 (1.8-2.1)	3.8 (3.3-4.4)	4.5 (3.9-5.1)	-
**Stroke**	3.3 (1.8-6.0)	3.1 (1.8-5.1)	2.9 (2.2-3.8)	2.0 (1.8-2.2)	3.4 (2.9-4.0)	3.6 (3.1-4.2)	<3.4

Grey =US database. Pink=South Korean database. Yellow=European database. SABA=short-acting beta-agonist. ARDS=acute respiratory distress syndrome. ARFS=acute renal failure syndrome. VThe=venous thromboembolic disease. PE=pulmonary embolism.

**Table 7.** Death within 30 days of COVID-19 diagnosis or hospitalization.

**Cohort**	**CU-AMC %** ** (95%CI)**	**CUIMC % ** **(95%CI)**	**Optum** **EHR %** **(95%CI)**	**VA-OMOP ** **% (95%CI)**	**HIRA % ** **(95%CI)**	**CPRD % ** **(95%CI)**	**IPCI %** **(95%CI)**	**SIDIAP % ** **(95%CI)**
**Diagnosed** **COVID-19**	11.6 (9.4-14.2)	18.8 (16.2-21.7)	3.7 (3.4-4.1)	8.3 (7.8-8.8)	-	24.9 (20.0-30.5)	23.5 (18.2-29.8)	15.6 (15.0-16.2)
**Hospitalized** ** COVID-19**	25.8 (21.2-31.0)	32.2 (28.0-36.7)	7.6 (6.9-8.4)	17.6 (16.5-18.7)	16.6 (11.4-23.5)	-	-	21.4 (20.3-22.6)

Grey =US database. Pink=South Korean database. Yellow=European database

## Discussion

COPD prevalence among patients with COVID-19 was 1.5- to 3-fold greater among those hospitalized than among those in the diagnosed cohort. Studies from China have reported the lowest observed COPD prevalence in COVID-19 patients with rates as low of 0.8% ^3^. In contrast, COPD prevalence appears greater in European and US COVID-19 study populations. We similarly observed a low COPD prevalence among COVID-19 patients from South Korea compared to other countries, which may reflect differences in the baseline prevalence of COPD in each country. It could also relate to differences in how health care systems responded to the pandemic, for example whether people with COPD were considered high risk and given advice on risk reduction measures and shielding.

COVID-19 patients with COPD in both cohorts had a similar age distribution with the proportion of men being consistently higher in the hospitalized cohort. Increasing age, male gender and a history of cardiovascular disease are established risk factors for severe COVID-19 ^14– 20^. We similarly observed a high prevalence of cardiometabolic comorbidities among COPD patients with COVID-19 from both cohorts. This includes arrythmia, which increases the risk from azithromycin and hydroxychloroquine use as treatment of COVID-19, particularly when prescribed in combination but also may be related to atrial fibrillation being prevalent within patients with COPD ^21, 22^.

Although exacerbation at presentation was recorded more commonly among hospitalized patients, overall exacerbation prevalence was relatively low. Further studies are required to formally assess to what degree typical exacerbations of COPD are a presenting feature of COVID-19 in people with COPD.

There have been safety concerns over the role of ICS with reports of worse COVID-19 outcomes associated with ICS use ^23, 24^. Whilst our study was not designed to formally assess this, we saw no large differences in ICS use between the cohorts as might be expected if use was associated with a large risk. Indeed, early clinical trials suggest that use of inhaled budesonide use may be beneficial ^25^. The increased use of acetaminophen, opioids and NSAIDs among hospitalized patients also suggests greater symptomatic illness. Whilst similar safety concerns with NSAID use have also emerged, recent studies have not found them to be harmful ^26, 27^.

The most common 30-day outcomes were ARDS, ARFS, arrhythmia, sepsis and heart failure suggesting that a multi-organ approach is required for COVID-19 clinical management. As expected, hospitalized COPD patients had a higher prevalence of poor health outcomes. However, it is useful to understand this risk among a cohort that includes milder cases at an earlier stage of the illness despite having similar levels of baseline comorbidity.

### Strength and limitations

A strength of this study is the federated analysis allowing large numbers of patients to be characterized between countries, which overcomes some of the limitations of smaller single centre studies and inevitable heterogeneity that can occur by applying different study designs and methods of analysis. Furthermore, information on a large number of additional patient characteristics relating to conditions and treatment are also available online beyond what has been presented here. The study has several limitations, however. First, the study is dependent upon the quality and extent of data captured by each database that could underestimate the prevalence of some characteristics. For conditions and treatments, the assumption was made that patients did not have the health condition or treatment if it were not captured in the database. A specific example of this is prior systemic corticosteroid use that ranged from 7.5% to 28.4% in the COVID-19 hospitalized cohort and 5.1% to 26.4% in the diagnosed cohort. The prevalence of systemic corticosteroid use was slightly higher than the prevalence of an exacerbation diagnosis. Whilst this suggests that some underestimation in exacerbation recording may have occurred, other indications for systemic corticosteroid in patients with COPD exist that could also explain the higher systemic corticosteroids use. Second, despite using a standardized data structure and method of analysis, heterogeneity between databases was still observed and it was not possible to determine whether this related to differences in clinical care compared to differences in the type of database. In this regard databases were a mixture between electronic health records and administrative claims capturing data from different health care settings. Lastly, our study was descriptive in nature with the aim of being hypothesis generating and informing healthcare resource use. It was not designed to examine causal associations or to establish causal inference in relation to differences in COVID-19 outcomes that may result from differences in patient characteristics such as comorbidity that would require specialized study designs and regression analyses. However, this type of evidence has still contributed to support the understanding COVID-19 in patients in many settings and could be useful as hypothesis generating for future studies in patients with COPD ^28^.

## Conclusions

COVID-19 patients with COPD are a vulnerable group with a high prevalence of other risk factors for severe COVID-19. No large differences in ICS use were seen between COPD patients with milder and more severe COVID-19 although further studies are required to confirm or refute this. COPD patients experience a high morbidity and mortality from COVID-19 and require a multi-organ approach to clinical management.

## Data availability

### Underlying data

Raw data from each database cannot be shared due to data privacy and governance requirements but raw data could be accessed according to the terms and conditions of each data source. The data source information including the terms and conditions for data access can be found in Table 8. Analyses were performed locally in compliance with all applicable data privacy laws. All aggregate data has been made freely available for public inquiry ( https://data.ohdsi.org/Covid19CharacterizationCharybdis/).

**Table 8.** Database information.

ID	Name	Description	Terms of Use	Terms and conditions for the access
CPRD	Clinical Practice Research Datalink	The Clinical Practice Research Datalink (CPRD) is a governmental, not-for-profit research service, jointly funded by the NHS National Institute for Health Research (NIHR) and the Medicines and Healthcare products Regulatory Agency (MHRA), a part of the Department of Health, United Kingdom (UK). CPRD consists of data collected from UK primary care for all ages. This includes conditions, observations, measurements, and procedures that the general practitioner is made aware of in additional to any prescriptions as prescribed by the general practitioner. In addition to primary care, there are also linked secondary care records for a small number of people. The major data elements contained within this database are outpatient prescriptions given by the general practitioner (coded with Multilex codes) and outpatient clinical, referral, immunization or test events that the general practitioner knows about (coded in Read or ICD10 or LOINC codes). The database also contains the patients’ year of births and any date of deaths.	1) Please allow for 2 weeks lead time for all publications using these results to go through internal review process. 2) The results are considered fit-for-use and were generated for this specific protocol. Derivations from the intent of this protocol are not validated by our institution.3) Our institution expects all authors to comply with all applicable personal data protection rules (such as the European Data Protection Regulation 2016/679, of April 27, 2016). 4) Our institution reserves the right to request to omit our results from a drafted publication if the findings could inflict reputational or institutional harm.	https://www.cprd.com/
CU-AMC HDC	U of Colorado Anschuz Medical Campus Health Data Compass (CU-AMC HDC)	Health Data Compass (HDC) is a multi-institutional data warehouse. HDC contains inpatient and outpatient electronic medical data including patient, encounter, diagnosis, procedures, medications, laboratory results from two electronic medical record systems (UCHealth and Children's Hospital of Colorado), state-level all-payers claims data, and the Colorado death registry. Acknowledgement statement: Supported by the Health Data Compass Data Warehouse project (healthdatacompass.org).	1) Please allow for 2 weeks lead time for all publications using these results to go through internal review process. 2) When using our results, you must always use this specific name when referring to our database. No other labels should be used in presenting our results. 3) The results are considered fit-for-use and were generated for this specific protocol. Derivations from the intent of this protocol are not validated by our institution. 4) Our institution reserves the right to request to omit our results from a drafted publication if the findings could inflict reputational or institutional harm.	https://www.healthdatacompass.org/
CUIMC	Columbia University Irving Medical Center	The clinical data warehouse of NewYork- Presbyterian Hospital/Columbia University Irving Medical Center, New York, NY, based on its current and previous electronic health record systems, with data spanning over 30 years and including over 6 million patients	Our institution reserves the right to request to omit our results from a drafted publication if the findings could inflict reputational or institutional harm. The results are specific to a study and should not be reused in other studies without review from our institution. For consistency, the Columbia database should be referred to as CUIMC.	https://www.cuimc.columbia.edu/ about-us/explore-cuimc/contact-cuimc gh13@columbia.edu
HealthVerity	HealthVerity	This HealthVerity derived data set contains de- identified patient information with an antibody and/or diagnostic test for COVID-19 linked to all available Medical Claims and Pharmacy Data from select private data providers participating in the HealthVerity marketplace.	1) Please allow for 2 weeks lead time for all publications using these results to go through internal review process. 2) The results are considered fit-for-use and were generated for this specific protocol. Derivations from the intent of this protocol are not validated by our institution.3) Our institution expects all authors to comply with all applicable personal data protection rules (such as the European Data Protection Regulation 2016/679, of April 27, 2016). 4) Our institution reserves the right to request to omit our results from a drafted publication if the findings could inflict reputational or institutional harm.	https://healthverity.com/license- healthcare-data-healthverity- marketplace/
HIRA	Health Insurance Review & Assessment Service	National claim data from a single insurance service from South Korea. It contains the observational medical records (including both inpatient and outpatient) of a patient while they are qualified to get the national medical insurance.	Review & Assessment service and the Ministry of Health and Welfare jointly release nationwide COVID- 19 patient’s de-identified data and do cooperation research together with the most prestigious academies and government organizations. Because raw data are owned in the organization so that cohort data are managed by result value sharing method with implementing analysis code without personal information leakage.	https://www.hira.or.kr/eng/main.do
IPCI	Integrated Primary Care Information	The Integrated Primary Care Information (IPCI) database is collected from EHR records of patients registered with 391 GPs throughout the Netherlands. The database contains records from approximately 2.6 million patients out of a Dutch population of 17M (8.2%) starting in 1996.	1) Results can only be used in the intent of a study that is approved by our governance board. Additional derived studies from large-scale analysis therefore require approval. 2) Inclusion of IPCI researchers is required for these derived studies to provide the proper context and interpretation of these results.	https://www.ipci.nl/
IQVIA-OpenClaims	IQVIA Open Claims	A United States database of open, pre-adjudicated claims from January 2013 to May 2020. Data are reported at anonymized patient level collected from office-based physicians and specialists via office management software and clearinghouse switch sources for the purpose of reimbursement. A subset of medical claims data have adjudicated claims.	Inclusion of IQVIA researchers is required in manuscripts using IQVIA data.	https://www.iqvia.com/solutions/real- world-evidence/real-world-data-and- insights
LPD-FRANCE	LPD FRANCE	LPD France is a computerised network of physicians including GPs who contribute to a centralised database of anonymised patient EMR. Currently, >1200 GPs from 400 practices are contributing to the database covering 7.8M patients in France. The database covers a time period from 1994 through the present. Observation time is defined by the first and last consultation dates. Drug information is derived from GP prescriptions. Drugs obtained over the counter by the patient outside the prescription system are not reported.	Inclusion of IQVIA researchers is required in manuscripts using IQVIA data.	https://www.iqvia.com/solutions/real- world-evidence/real-world-data-and- insights
LPDItaly	IQVIA LPD Italy	LPD Italy is comprised of anonymised patient records collected from software used by GPs during an office visit to document patients’ clinical records. Data coverage includes over 2M patient records with at least one visit and 119.5M prescription orders across 900 GP practices. Dates of service include from 2004 through present. Observation time is defined by the first and last consultation dates. Drugs are captured as prescription records with product, quantity, dosing directions, strength, indication and date of consultation.	Inclusion of IQVIA researchers is required in manuscripts using IQVIA data.	https://www.iqvia.com/solutions/real- world-evidence/real-world-data-and- insights
OptumEhr	Optum© de-identified Electronic Health Record Dataset	Optum© de-identified Electronic Health Record Dataset is derived from dozens of healthcare provider organizations in the United States (that include more than 700 hospitals and 7,000 Clinics treating more than 103 million patients) receiving care in the United States. The medical record data includes clinical information, inclusive of prescriptions as prescribed and administered, lab results, vital signs, body measurements, diagnoses, procedures, and information derived from clinical Notes using Natural Language Processing (NLP).	1) Please allow for 2 weeks lead time for all publications using these results to go through internal review process. 2) The results are considered fit-for-use and were generated for this specific protocol. Derivations from the intent of this protocol are not validated by our institution.3) Our institution expects all authors to comply with all applicable personal data protection rules (such as the European Data Protection Regulation 2016/679, of April 27, 2016). 4) Our institution reserves the right to request to omit our results from a drafted publication if the findings could inflict reputational or institutional harm.	https://www.optum.com/business/ solutions/life-sciences/real-world-data /ehr-data.html ?
SIDIAP	Information System for Research in Primary Care (SIDIAP)	The Information System for Research in Primary Care (SIDIAP; www.sidiap.org) is a primary care records database that covers approximatly 80% of the population of Catalonia, North-East Spain. Healthcare is universal and tax-payer funded in the region, and primary care physicians are gatekeepers for all care and responsible for repeat prescriptions.	1) When using our results, you must always use this specific name and this citation when referring to our database. No other labels should be used in presenting our results: Information System for Research in Primary Care (SIDIAP). 2) The results are considered fit-for-use and were generated for this specific protocol. Derivations from the intent of this protocol are not validated by our institution. 3) Our institution expects all authors to comply with all applicable personal data protection rules (such as the European Data Protection Regulation 2016/679, of April 27, 2016). 4) Our institution reserves the right to request to omit our results from a drafted publication if the findings could inflict reputational or institutional harm.	https://www.sidiap.org/index.php/en
STARR- OMOP	STARR-OMOP	STAnford medicine Research data Repository, a clinical data warehouse containing live Epic data from Stanford Health Care, the Stanford Children’s Hospital, the University Healthcare Alliance and Packard Children's Health Alliance clinics and other auxiliary data from Hospital applications such as radiology PACS. STARR platform is developed and operated by Stanford Medicine Research IT team and is made possible by Stanford School of Medicine Research Office. https://arxiv.org/abs/2003.10534	1) When using our results, you must always use this specific name and this citation when referring to our database. No other labels should be used in presenting our results. 2) The results are considered fit-for-use and were generated for this specific protocol. Derivations from the intent of this protocol are not validated by our institution. 3) Our institution expects all authors to comply with all applicable personal data protection rules 4) Our institution reserves the right to request to omit our results from a drafted publication if the findings could inflict reputational or institutional harm.	https://med.stanford.edu/starr-omop. html
VA-OMOP	Department of Veterans Affairs	VA OMOP data reflects the national Department of Veterans Affairs health care system, which is the largest integrated provider of medical and mental health services in the United States. Care is provided at 170 VA Medical Centers and 1,063 outpatient sites serving more than 9 million enrolled Veterans each year.	1) Please allow for 2 weeks lead time for all publications using these results to go through internal review process. 2) When using our results, you must always use this specific name and this citation when referring to our database. No other labels should be used in presenting our results. We would like to have the name and description of the database standardized.3) The results are considered fit-for-use and were generated for this specific protocol. Derivations from the intent of this protocol are not validated by our institution. 4) Our institution expects all authors to comply with all applicable personal data protection rules (such as the European Data Protection Regulation 2016/679, of April 27, 2016). 5) Our institution reserves the right to request to omit our results from a drafted publication if the findings could inflict reputational or institutional harm. In line with item 3, we would like to make sure that data created and validated with one use case in mind still fits for other use cases. We do not anticipate examples where data would produce such harm (outside of some data quality issue / need for retraction), but if that were the case, we would need to alert VA leadership and ensure the wording was objective. 6) We need to acknowledge our funding using language like: "This work was supported using resources and facilities of the Department of Veterans Affairs (VA) Informatics and Computing Infrastructure (VINCI), VA HSR RES 13–457." This can be shortened and arranged in the acknowledgement section with others. 7) We need a disclaimer such as: "The views expressed are those of the authors and do not necessarily represent the views or policy of the Department of Veterans Affairs or the United States Government." This can be shorted and combined with other institutions' disclaimers.	https://www.data.va.gov/

All analytic code and result sets have been made available ( https://github.com/ohdsi-studies/Covid19CharacterizationCharybdis).

Archived analysis code as at time of publication: https://doi.org/10.5281/zenodo.5779264 ^29^. Code is available under the terms of the Apache License 2.0.

### Extended data

Zenodo: Characteristics and outcomes of COVID-19 patients with COPD from the United States, South Korea, and Europe - Supplementary Materials. https://doi.org/10.5281/zenodo.5729423 ^11^.

This project contains the following extended data:

- **Supplementary Table S1**. Age and gender distribution of hospitalized COVID-19 patients with COPD.
- **Supplementary Table S2**. Age and gender distribution of diagnosed COVID-19 patients with COPD.
- **Supplementary Table S3**. Prevalence of treatments in patients with COPD in the 30 days before COVID-19 diagnosis.
- **Supplementary Table S4**. Prevalence of outcomes in diagnosed COVID-19 patients with COPD with 95%CI.
- **Supplementary Figure S1**. Flow chart showing database selection.
- **Supplementary Figure S2**. Prevalence of age and gender among COPD patients with COVID-19 who have been diagnosed and hospitalized.
- **Supplementary Figure S3**. Comparison of characteristics between COPD patients with COVID-19 in the diagnosed and hospitalized cohorts by SMD.
- **Appendix 1**. Overview of Data Sources Screened for Eligibility and Contributing Results
- **Appendix 2**. Definitions and codes used to identify COVID-19
- **Appendix 3**. Definitions and codes used to identify COPD patients

Data are available under the terms of the Creative Commons Attribution 4.0 International Public License (Attribution 4.0 International).
