# Supplementary material for: Characteristics and outcomes of COVID-19 patients with COPD from the United States, South Korea, and Europe
Source: Wellcome Open Res. 2023 Jan 10;7:22. Originally published 2022 Jan 24. [Version 3] doi: 10.12688/wellcomeopenres.17403.3 (PMC9951545; doi:10.12688/wellcomeopenres.17403.3)
Supplement: Peer review history

# Peer review: Reviewer response for version 3

**DOI:** 10.21956/wellcomeopenres.20813.r54044

**Published:** 2023-01-13

**Authors:** Laurie Tomlinson

**Affiliations:** 1 Department of Non-Communicable Disease Epidemiology, London School of Hygiene & Tropical Medicine, London, UK

The authors have addressed the minor issues I commented on previously - many thanks.

Is the work clearly and accurately presented and does it cite the current literature?

Partly

If applicable, is the statistical analysis and its interpretation appropriate?

Yes

Are all the source data underlying the results available to ensure full reproducibility?

Partly

Is the study design appropriate and is the work technically sound?

Yes

Are the conclusions drawn adequately supported by the results?

Yes

Are sufficient details of methods and analysis provided to allow replication by others?

Yes

Reviewer Expertise:

Pharmacoepidemiology and clinical epidemiology

I confirm that I have read this submission and believe that I have an appropriate level of expertise to confirm that it is of an acceptable scientific standard.

# Peer review: Reviewer response for version 2

**DOI:** 10.21956/wellcomeopenres.19703.r52973

**Published:** 2022-12-14

**Authors:** Laurie Tomlinson

**Affiliations:** 1 Department of Non-Communicable Disease Epidemiology, London School of Hygiene & Tropical Medicine, London, UK

This is a powerful multi-database, multi-country study that carefully describes the demographics of COVID-19 patients with COPD during the first wave of the pandemic. It carefully describes the data sources and methods, and does not over interpret the causal significance of their findings.

 I have a few minor points that I think can be easily addressed.

1. I think the definition of hospital cases will include those who caught COVID-19 while in hospital. These people tended to have a different disease course and may not represent the patients who were admitted with COVID-19. This could be pointed out as a limitation.
2. There is likely to be some misclassification of asthmatics who were classified through medication use and were asthma had not been coded. These people may have fewer comorbidities than those with COPD: again this is simply a minor limitation.
3. The section about hydroxychloroquine and azithromycin is now historic and could be removed – or clarified that this will only relate to a small % of people in analyses of Wave 1.
4. A key limitation is the differences in prevalence and timing of Wave 1 in these countries in relation to the period of data capture, as well as the healthcare response in each country. This is likely to impact on between country differences, particularly mortality. This should be highlighted at least in the discussion.

Is the work clearly and accurately presented and does it cite the current literature?

Partly

If applicable, is the statistical analysis and its interpretation appropriate?

Yes

Are all the source data underlying the results available to ensure full reproducibility?

Partly

Is the study design appropriate and is the work technically sound?

Yes

Are the conclusions drawn adequately supported by the results?

Yes

Are sufficient details of methods and analysis provided to allow replication by others?

Yes

Reviewer Expertise:

Pharmacoepidemiology and clinical epidemiology

I confirm that I have read this submission and believe that I have an appropriate level of expertise to confirm that it is of an acceptable scientific standard, however I have significant reservations, as outlined above.

# Peer review: Reviewer response for version 2

**DOI:** 10.21956/wellcomeopenres.19703.r49458

**Published:** 2022-03-31

**Authors:** Marta A. Kisiel

**Affiliations:** 1 Department of Medical Sciences, Environmental and Occupational Medicine, Uppsala University, Uppsala, Sweden

I have read the current version of manuscript and it is improved, the authors responded to all my questions. I have no more questions at this moment and I would recommend the manuscript for indexing.

Is the work clearly and accurately presented and does it cite the current literature?

Yes

If applicable, is the statistical analysis and its interpretation appropriate?

Yes

Are all the source data underlying the results available to ensure full reproducibility?

Partly

Is the study design appropriate and is the work technically sound?

Partly

Are the conclusions drawn adequately supported by the results?

Yes

Are sufficient details of methods and analysis provided to allow replication by others?

Partly

Reviewer Expertise:

NA

I confirm that I have read this submission and believe that I have an appropriate level of expertise to confirm that it is of an acceptable scientific standard.

# Peer review: Reviewer response for version 1

**DOI:** 10.21956/wellcomeopenres.19241.r48211

**Published:** 2022-01-31

**Authors:** Marta A. Kisiel

**Affiliations:** 1 Department of Medical Sciences, Environmental and Occupational Medicine, Uppsala University, Uppsala, Sweden

This study addressed an important topic, investigating COVID-19 patients with COPD. The study is based on several databases from different countries. The main conclusion was that there were more COPD patients in the hospitalised COVID-19 group than in the diagnosed COVID-19 group. The authors highlighted the limitation of heterogenicity of the cohorts. Also, the accuracy of COPD diagnosis in all included cohorts might be a limitation that should be added. The authors show other comorbidities like obesity, cardiovascular diseases and diabetes. It might be that association of COPD and hospitalised COVID-19 should be adjusted by other comorbidities. 

 The information on different cohorts is hard to find among all supplementary tables and might be organised in a more available form. As the authors should extend the list of study limitations. 

 The study should undergo a minor revision before it can be accepted. 

Is the work clearly and accurately presented and does it cite the current literature?

Yes

If applicable, is the statistical analysis and its interpretation appropriate?

Yes

Are all the source data underlying the results available to ensure full reproducibility?

Partly

Is the study design appropriate and is the work technically sound?

Partly

Are the conclusions drawn adequately supported by the results?

Yes

Are sufficient details of methods and analysis provided to allow replication by others?

Partly

Reviewer Expertise:

Asthma, Occupational Lung Disease, Smal airways, Post covid

I confirm that I have read this submission and believe that I have an appropriate level of expertise to confirm that it is of an acceptable scientific standard, however I have significant reservations, as outlined above.

# Response

**Published:** 2022-03-18

**Authors:** David Moreno Martos

**Affiliations:** University of Dundee, UK

We thank the reviewer for their time in reviewing the manuscript. We agree it is entirely possible that some of the differences in outcomes between COVID-19 diagnosed and hospitalised patients may be as a result of other comorbidities. However, this study is descriptive in nature and hypothesis generating rather than intended to provide causal inference, which would be beyond the scope of the current study. We have now expanded the study limitations in the Discussion. Journal requirements preclude us to include supplementary material here although we have re-formatted the supplementary material document to improve clarity.
